# *Bacillus species* (BT42) isolated from *Coffea arabica* L. rhizosphere antagonizes *Colletotrichum gloeosporioides* and *Fusarium oxysporum* and also exhibits multiple plant growth promoting activity

**DOI:** 10.1186/s12866-016-0897-y

**Published:** 2016-11-18

**Authors:** Tekalign Kejela, Vasudev R. Thakkar, Parth Thakor

**Affiliations:** 1 Department of Biology, Faculty of Natural and Computational Sciences, Mettu University, Mettu, Ethiopia; 2 Present Address: BRD school of Biosciences, Sardar Patel University, Vallabh Vidyanagar, 388120 India; 3 BRD School of Biosciences, Sardar Patel University, Vadtal Road, Satellite Campus, Post Box No.39, Vallabh Vidyanagar, 388120 Gujarat India

**Keywords:** Biocontrol, *Colletotrichum gloeosporioides*, *Fusarium oxysporum*, Plant growth promoting rhizobacteria, *Coffea arabica* L

## Abstract

**Background:**

*Colletotrichum* and *Fusarium* species are among pathogenic fungi widely affecting *Coffea arabica* L., resulting in major yield loss. In the present study, we aimed to isolate bacteria from root rhizosphere of the same plant that is capable of antagonizing *Colletotrichum gloeosporioides* and *Fusarium oxysporum* as well as promotes plant growth.

**Results:**

A total of 42 *Bacillus* species were isolated, one of the isolates named BT42 showed maximum radial mycelial growth inhibition against *Colletotrichum gloeosporioides* (78%) and *Fusarium oxysporum* (86%). BT42 increased germination of *Coffee arabica* L. seeds by 38.89%, decreased disease incidence due to infection of *Colletotrichum gloeosporioides* to 2.77% and due to infection of *Fusarium oxysporum* to 0 (*p* < 0.001). The isolate BT42 showed multiple growth-promoting traits. The isolate showed maximum similarity with *Bacillus amyloliquefaciens*.

**Conclusion:**

*Bacillus species* (BT42), isolated in the present work was found to be capable of antagonizing the pathogenic effects of *Colletotrichum gloeosporioides* and *Fusarium oxysporum*. The mechanism of action of inhibition of the pathogenic fungi found to be synergistic effects of secondary metabolites, lytic enzymes, and siderophores. The major inhibitory secondary metabolite identified as harmine (β-carboline alkaloids).

## Background

The word coffee comes from the name of the place in Ethiopia called “Kaffa”. “Kaffa” means the plants of God [1]. Coffee classified under the family of *Rubiaceae* in the genus *Coffea*. There are many species of coffee, but the two most widely cultivated are *C. arabica* L. and *C. canephora (robusta)*. Southwestern and southeastern Ethiopia considered as the origin of *C. arabica* L. (Arabica coffee) [2]. Of the total world production of coffee, *C. arabica* L. takes the lion’s share, which is 66% and *C. canephora* only of 34%. Although coffee produced in few countries, it is the most traded agricultural products around the globe after oil. According to a 2014 report by the International Coffee Organization, the top six coffee producing countries in our globe are Brazil, Vietnam, Colombia, Indonesia, Ethiopia, and India. In Ethiopia, it is mostly exported cash crop that accounts for 69% of all agriculturally export commodities and it was estimated that at least 15 million of Ethiopian population depend directly or indirectly on coffee production [1]. Similarly, there are around 250,000 coffee growers in India; 98% of them are small-scale growers.

One of the challenges in coffee production industry is the impact of the coffee pathogen, especially pathogenic fungi, which results in reduced production and low quality of coffee seeds [3]. The yield loss due to the fungal pathogens, especially *Colletotrichum species,* and *Fusarium species* have been repeatedly reported from coffee growing areas [4–7]. Coffee berry disease caused by *Colletotrichum khawae* is causing a major yield loss in coffee growing areas of Ethiopia [8, 9]. *C. khawae* and *C. gloeosporioides* are the most abundantly found pathogens in diseased coffee seeds [9]. *C. gloeosporioides* also listed as one of important coffee pathogens by the coffee board of India. Apart from the coffee plant, *C. gloeosporioides Penz* possesses a broad host range (470 genera of plants) and ranked as the most devastating plant pathogen in the genus *Colletotrichum* [10, 11]. *Fusarium species* also cause serious impact on the coffee production industry. Coffee wilt disease caused by *Gibberella xylarioides* (anamorph: *Fusarium xylarioides*) causes approximately 3360 t of coffee yield losses each year in Ethiopia [12]. This production loss causes a great economic loss around the world, for example, Ethiopia loses an estimated 3.7 million American dollars every year.

Chemical pesticides used currently to control coffee pathogens needed to spray 7–8 times annually which is laborious and expensive. Furthermore, the extensive use of chemical pesticides also contributes to emerging pesticide resistant pathogens. The uses of chemical pesticides and fertilizers have also a negative impact on the indigenous microbial community by disturbing the natural distribution of microbial niche. The coffee cultivated with no or less application of chemical pesticides have more consumer acceptance. In addition, the use of environmentally friendly and sustainable way of disease controlling system gained major attention in recent years. In this view, rhizosphere is the ideal place to search potential rhizobacteria that are capable of promoting plant growth and suppressing the phytopathogens.

Extensive studies of the use of plant growth promoting rhizobacteria (PGPR) for disease control and plant growth promotion in the coffee plant have not been reported. It is necessary and useful to evaluate and document indigenous beneficial microbe isolated from coffee and test them against coffee pathogens. In the current study, several bacteria from the rhizosphere of *C. arabica* L. were isolated and the potent bacterium antagonistic to *C. gloeosporioides and F. oxysporum was* chosen, which also showed multiple plant growth promoting activity. The potent isolate showed maximum similarity with *Bacillus amyloliquefaciens* by 16 s rRNA gene sequencing and by blasting this sequence against reference sequences found in the international nucleotide database using the program called BLASTn.

## Results

### Isolation of Bacillus species

**Fig. 1:**
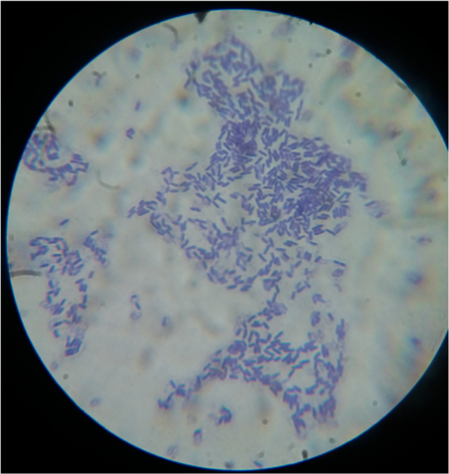
Gram staining of cells of *Bacillus sp* BT42 isolated from *Coffea arabica* L. rhizosphere

From the rhizosphere of *Coffea arabica* L. 42 pure *Bacillus species* were isolated. These isolates were gram-positive, catalase-positive, spore-forming, rod-shaped and able to survive at 80 °C (Fig. 1). It forms central/sub terminal/ellipsoidal endospores. The bacterium grew at the temperature range of 15–50 °C, the optimum temperature of 30–42 °C, optimum pH of 7 in aerobic condition.

### The antagonistic effect of Bacillus species isolates against C. gloeosporioides and F. oxysporum

**Fig. 2:**
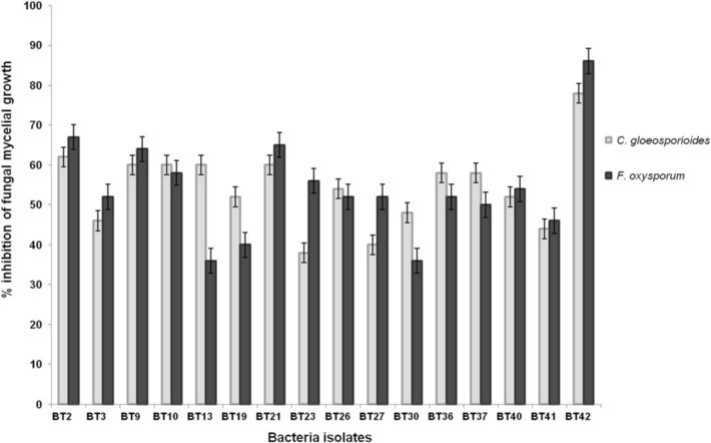
In vitro mycelial growth inhibition of *C. gloeosporioides* and *F. oxysporum* by *Bacillus species* isolated from *Coffea arabica* L. rhizosphere. Error bars represent ± Standard Deviation (SD). Values are means of three replicates

Bacteria isolates were studied against two fungal pathogens *C. gloeosporioides* and *F. oxysporum* for radial mycelial growth inhibition. Sixteen *Bacillus* species isolates showed greater than 40% mycelial growth inhibition. Among these isolates, BT42 showed maximum radial mycelial growth inhibition against *C. gloeosporioides* (78%) and *F. oxysporum* (86%) (Fig. 2). Therefore, BT42 selected for in vitro and vivo studies.

### Identification of isolate

**Fig. 3:**
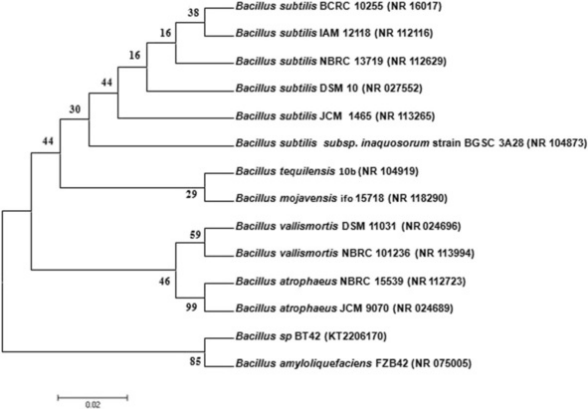
Neighbor joining tree of isolated *Bacillus* sp. BT42 and closely related species. Bootstrap values based on 1,000 replications

The isolate BT42 selected for identification because it showed highest mycelial growth inhibition of *C. gloeosporioides* and *F. oxysporum* when compared to other isolates. The 16S rRNA gene amplified from the genomic DNA of BT42 (1.5 kb) sequenced and analyzed by nucleotide Blast analysis (BLASTn). The BT42 (NCBI accession number KT220617) showed maximum similarity with *Bacillus amyloliquefaciens* (Fig. 3). When compared to Ez taxon database it showed 86.55% similarity with *Bacillus amyloliquefaciens* subsp. plantarum FZB42.

### Plant growth promoting characteristics of Bacillus species BT42

**Fig. 4:**
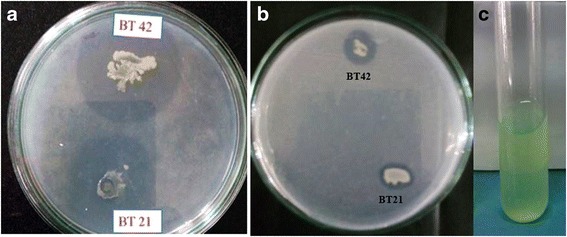
Zinc solubilization (**a**), Tricalcium phosphate solubilization (**b**) and Siderophore production (**c**) by Bacillus species(BT42)

BT42 produced 14.56 ± 0.862 μg/ml IAA in the medium supplemented with L-tryptophan, produced ammonia, solubilized 6.36 ± 0.48 μg/ml tri-calcium phosphate, formed 37.5 ± 0.56 mm of holo zone by solubilizing insoluble zinc oxides (Fig. 4), produced 75.90 ± 1.24% siderophore units in the iron free succinate medium, grew on the media supplemented with 1-aminocyclopropane-1-carboxylic acid (ACC) as a sole nitrogen source, and formed robust pellicles when grown in LB broth at the liquid air interference.

### Bioassay

#### Effect of BT42 on seed germination and disease incidence

**Fig. 5:**
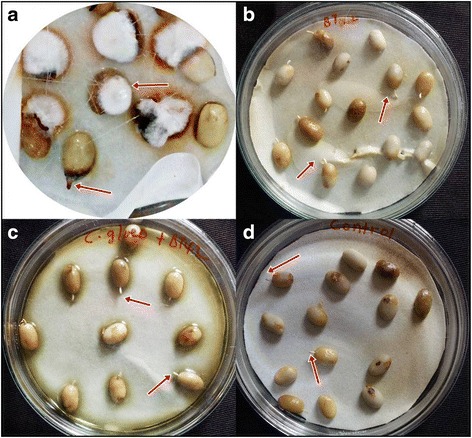
Effect of inoculation of BT42 on coffee seed germination. *C. gloeosporioides* infected (**a**), BT42 treated (**b**), BT42+ *C. gloeosporioides* (**c**) and untreated (**d**)

To study the effect of BT42 on germination of *C. arabica* L. seeds, suspension of overnight grown rhizobacteria (0.5 McFarland Standard) was coated on the seeds of *C. arabica L* and the effect on germination were observed and recorded. Percentage germination of *C. arabica L.* improved from 50% of untreated seeds to higher in the presence of rhizobacteria (Table 1). None of the *C. arabica L.* seeds infected with *C. gloeosporioides* could germinate (Fig. 5a), but in the presence of BT42, there was an improvement in germination percentage and decrease in the disease incidence. The disease incidence was as high as 91.67% when the seeds infected by *C. gloeosporioides* spore suspension alone. Disease incidence reduced by 88.9% when the BT42 was simultaneously surface coated with *C. gloeosporioides* (Table 1).

**Table 1:** Effects of selected rhizobacteria isolates on germination of *C. arabica* L. seeds and on disease incidence caused by *C. gloeosporioides* and *F. oxysporum*

Bioassay	Parameters	
	Germination %	DI (%)
Control (untreated seeds)	50 ± 5.33^a^	0^a^
*C. gloeosporioides* infected	0^b^	91.67 ± 8.33^b^
BT42 treated	88.89 ± 4.81^c^	0^a^
BT42 + *C. gloeosporioides*	72.22 ± 2.83^d^	2.77 ± 4.81^a^
Control (untreated seeds)	50 ± 5.33^a^	0^a^
*F. oxysporum* infected seeds	11.11 ± 4.81^b^	88.89 ± 9.62^b^
BT42 treated	88.89 ± 4.81^c^	0^a^
BT42 + *F. oxysporum*	80.56 ± 12.72^c^	0^a^

Values followed by dissimilar letters in each column indicate significance difference (one-way ANOVAs, Duncan’s test). Values are means of three replicates

### Mechanisms of inhibition

#### Lytic enzyme production

**Fig. 6:**
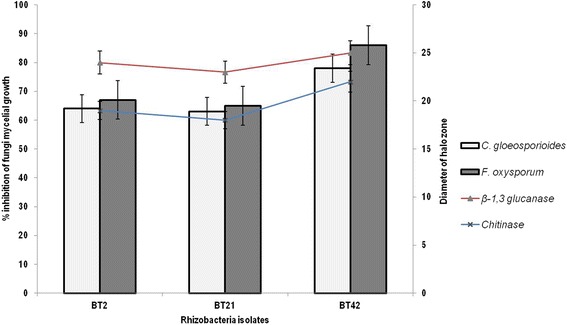
Percent inhibition of mycelial growth of *C. gloeosporioides* and *F.oxysporum* (*Bar graph*) and production of lytic enzymes (*line graph*) by selected *Bacillus species* isolates

BT42 formed a clear zone around its colony on the medium supplemented with colloidal chitin, laminarin, tween 80 and skim milk, indicating extracellular production of chitinase, β-1,3 glucanase, protease, and lipase respectively. The correlation of diameter of hole zone of chitinase with mycelial growth inhibition of *C. gloeosporioides* (*r* = 0.905, *P* < 0.05) and *F. oxysporum* (*r* = 0.780, *P* < 0.05) were positive. Similarly, the correlation of diameter of hole zone of β-1,3 glucanase with mycelial growth inhibition of *C. gloeosporioides* (*r* = 0.604, *P* < 0.01) and *F. oxysporum* (*r* = 0.802, *P* < 0.01) were also positive (Fig. 6).

#### Production of Antifungal compounds

**Fig. 7:**
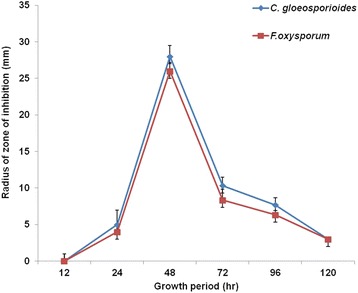
The radius of inhibition of pathogenic fungi by metabolite extracted from BT42 at different period of growth. Error bar represents ± SD

To investigate the production of antifungal metabolite/s, 5 ml of growing culture of BT42 (in three replicates) was taken at different time intervals and its supernatant was tested for antifungal activity against *C. gloeosporioides* and *F. oxysporum* by agar well diffusion method. The highest antifungal activity was found in 48 h old culture grown at 30 °C under shaking condition of 150 rpm (Fig. 7).

#### Stability of antifungal compounds

The ethyl acetate extract of 48-hour-old cell-free culture was checked for its antifungal activity at different temperatures and it was found stable for more than 3 months at 4 °C, for 1 h at 100 °C and for 3 months at room temperature.

#### Separation and purification of antifungal compound

**Fig. 8:**
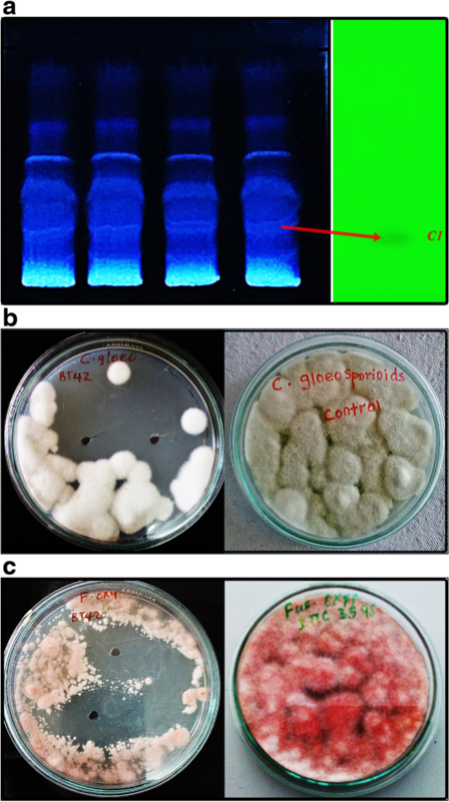
TLC showing separation of extract and purified band of C1 (**a**), inhibition of growth of *C. gloeosporioides* by C1 (**b**) inhibition of growth of *F. oxysporum* by C1 (**c**)

The ethyl acetate extract was separated on the silica thin layer chromatography (TLC) plate using isopropanol: ammonia: water (10:1.5:1) as the solvent system (Fig. 8a). Five bands with R*f* values 0.33, 0.50, 0.61, 0.72 and 0.87 were detected. Compounds corresponding to a metabolite in each band named from bottom to top as C1, C2, C3, C4, and C5 were assayed for antifungal activity against *C. gloeosporioides* and *F. oxysporum*. Among the five bands, the metabolite named as C1 showed significant mycelial growth inhibition of *C. gloeosporioides* (Fig. 8b) and *F. oxysporum* (Fig. 8c) and was selected further for characterization and identification. The purity of the band showing maximum antifungal activity (that is C1, *Rf* 0.33) was checked again using different solvent systems on TLC, which showed a single band.

#### Characterization and identification of potent compound

**Fig. 9:**
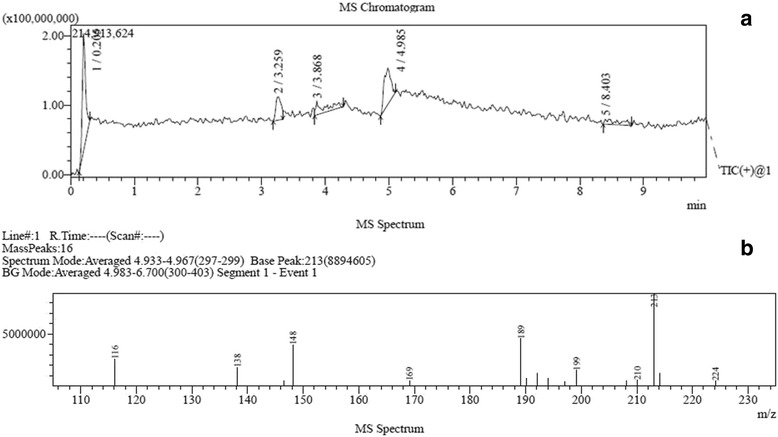
**a** Chromatogram based on the retention time in the LC-MS. **b** Mass spectrum of the peak 4 having retention time 4.9

**Fig. 10:**
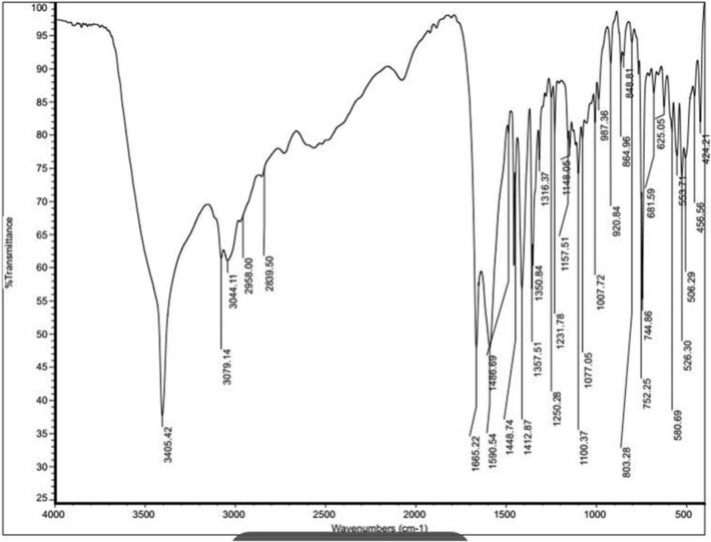
FTIR data with the presence of functional groups in the potent compound C1, frequency values appeared in the figures represent the respective functional groups

**Fig. 11:**
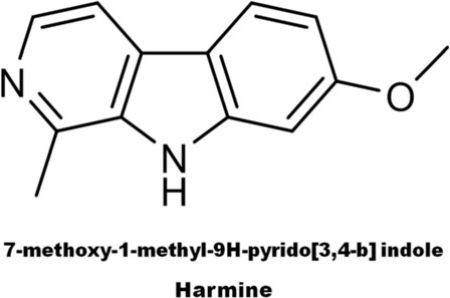
Structural formula and name of potent compound C1

Purified compound C1 was subjected to spectral scan analysis from 100–1100 nm to identify its absorbance maxima, which was found to be 205 nm. The compound C1 was found to be soluble in methanol and sparingly soluble in water. Liquid chromatography-mass spectrometry (LC-MS) data of C1 showed the retention time 4.9 min by the photodiode anode (PDA) detector. The same fragment was subjected to mass spectrometry (MS) analysis. MS analysis clearly indicated that purified C1 compound has the molecular weight 212.10 (Fig. 9a). For the investigation of the numbers of carbon, hydrogen, oxygen and nitrogen atoms, absolute intensity of M + 1 peak and natural abundance of isotopes were considered. Based on the calculation we found that there were 13 carbons, 12 hydrogens, 2 nitrogens and 1 oxygens atom. From m/z cloud the value of mass (i.e. 212.09) (Fig. 9b) is exactly matching with the compound harmine (CAS registry Number. 442-51-3) and its MS spectrum was matched to NIST database. Fourier transform infrared spectroscopy (FTIR) data of the compound C1 showed the presence of functional groups. The frequency at 3405 cm^-1^ indicates the presence of -NH Indole stretching, while aromatic C-H stretching at 3044 cm^-1^, -CH of alkane stretching at 2958 cm^-1^, C = N stretching at 1665 cm^-1^, -CH of -CH_3_ bending at 1357 cm^-1^, asymmetrical C-O-C stretching at 1077 and 1231 cm^-1^, C-N of Indole at 1100 cm^-1^, aromatic C = C stretching at 1590 and 1448 cm^-1^, -CH bending of aromatic ring at 864 cm^-1^ were observed (Fig. 10). Based on LC-MS data and FTIR assignment, the suggested formula for the compound is C_13_H_12_N_2_O and its structural formula showed in Fig. 11.

Further confirmation of the compound was carried out by qualitative tests using the general reagent for alkaloids (Dragendroff’s reagent) and it was found to be positive and specifically for the presence of indole derivates using glyoxylic-sulphuric acid test. Accordingly, the solution containing the C1 showed a positive test for the presence of alkaloid and showed a positive test for the presence of indole derivative, forming a purple to violet ring at the junction of two distinct phases when glyoxylic acid and the test solution mixed in the presence of conc. H_2_SO_4_. The secondary metabolite harmine could be the principal reason for inhibition of growth of *C. gloeosporioides* and *F. oxysporum*.

## Discussion

In the present study, we have isolated potent *Bacillus species* (BT42) that is able to inhibit C*. gloeosporioides* (78%) and *F. oxysporum* (86%). In a review of similar studies, *Bacillus sp*. strain RMB7, which has broad range antifungal activity showed 71% and 78% mycelial growth inhibition of *F. oxysporum* and *C. gloeosporioides* respectively [13]. BT42 produces known extracellular lytic enzymes (chitinase and β-1, 3-glucanase), which are significantly correlated with fungi mycelial growth inhibition. In a similar study conducted elsewhere, significant mycelial growth inhibition of *G. xylarioides* (anamorph: *Fusarium xylarioides*) by the chitinase producing *Bacillus* species isolate named as JU5444 was also reported [12]. Apart from chitinase and β-1,3-glucanase, BT42 produced protease and lipase that might be involved in the inhibition of the mycelial growth of *C. gloeosporioides and F. oxysporum*. Earlier reports indicated that protease [14, 15] and lipase production [12] by bacteria are associated with inhibition of mycelial growth of different fungi.

Since the statistical correlation of growth inhibition of pathogenic fungi and lytic enzyme produced were positive but mostly less than 0.9 r, more than one mechanism of inhibition of pathogenesis was suspected. The production of siderophore by BT42 might also be involved in the inhibition of fungi. Bacteria produce Siderophores during iron starvation. The previous report indicates that siderophores produced by *Bacillus subtilis* have got potential to inhibit the growth of *F. oxysporum* [16]. *Bacillus amyloliquefaciens* FZB42, which has maximum similarity with BT42, harbors genes responsible for the synthesis of siderophores [17].

Apart from production of lytic enzymes and siderophores, from the culture filtrate of BT42, we also isolated and identified antifungal compound, which is confirmed as Harmine [18]. Harmine is β-carboline alkaloid that was first isolated from *Peganum harmala* L [19, 20]. Harmine is rarely reported from bacteria and it is mostly extracted from higher plants [19, 21]. Harmine production in bacteria was limited to a few species including *Enterococcus faecium, Myxobacter* and *Pseudomonas* species [22–25]. However, the recent report by Saad and Zakaria demonstrated that *Bacillus flexus* isolated from fresh water produced harmine as a mechanism of inhibition of toxic cyanobacteria and this was considered as the first report from *Bacillus* species [26]. In our present study, we identified harmine from the cultural extract of *Bacillus species* (BT42) isolated from root rhizosphere of *C. arabica* L., which is the first report of its type. This is the first report of inhibition of pathogens of *C. arabica* L., *C. gloeosporioides* and *F. oxysporum* by plant growth promoting rhizobacterium by an alkaloid harmine. The inhibitory activity of β-carboline alkaloids against different fungi that include *C. gloeosporioides* and *F. oxysporum* was recently described although the β-carboline alkaloids were extracted from the plant [21].

To sum up, the highest inhibition of *C. gloeosporioides and F. oxysporum* mycelial growth by the *Bacillus species* (BT42) is due to the synergistic effect of multiple mechanisms, which could be explained as the production of lytic enzymes and harmine, which is produced as an extracellular secondary metabolite.

Apart from biocontrol activity, *Bacillus species* (BT42) also exhibited plant growth promoting characteristics (IAA production, ammonia production, phosphate solubilization and zinc solubilization) that directly involved in plant growth promotion and indirectly in the pathogen suppression. The production of IAA by rhizobacteria is so important in supporting plant growth and development by interfering with the endogenous IAA produced by the plant and serve in defense responses [27–29]. Studies showed that *Bacillus amyloliquefaciens* is known to produce a substantial amount of IAA [17, 30]. Another important trait displayed by *Bacillus species* (BT42) is the ability to produce an enzyme ACC deaminase. The production of ACC deaminase by the BT42 is also vital in plant-microbe interaction; that enables the host plant to withstand stress due to drought and flooding by decreasing the endogenous ethylene levels. Studies showed that *Bacillus amyloliquefaciens* has the versatile potential for both plant growth and biocontrol activity of different phytopathogenic fungi, which is similarly strengthened by our present study [17, 31–33].

The high disease incidence caused by *C. gloeosporioides* and *F. oxysporum* were significantly reduced in the presence of BT42 due to the potential biocontrol activity of this bacterium. In BT42 treated *C. arabica* L seeds, germination occurred much better than that of untreated, which indicates that the isolate facilitated germination of the *C. arabica* L. seeds by the extracellular production of PGPR traits that supported the germination.

## Conclusions

In conclusion, we have isolated a *Bacillus species* (BT42), which shows the versatility of direct and indirect plant growth promoting traits. BT42 showed maximum similarity with *Bacillus amyloliquefaciens* in NCBI BLASTn result and shows maximum similarity with the same bacterium by Ez taxon database. This bacterium could effectively inhibit pathogens of *C. arabica* L*.* The mechanisms of inhibition of *C. gloeosporioides* and *F. oxysporum* were found to be the production of lytic enzymes, siderophores as well as antifungal compounds. The major antifungal compound was identified as harmine (a member of β-carboline alkaloids), which is not previously reported as a mechanism of action of PGPR. To this end, the BT42 isolate can be a potential candidate to be used as a biocontrol of *C. gloeosporioides* and *F. oxysporum* and also as biofertilizer.

## Methods

### Sampling site and sample collection

Rhizosphere soil samples of *Coffea arabica* L. were collected from two different fields at Khusalnagar, Karnataka, South India. These sites are located between 12^0^28’05.7”N and 75^0^57’51.7”E. Soil samples were collected after consent was obtained from coffee farm owners.

### Isolation and Identification of bacterial isolate

Isolation of rhizobacteria from root rhizosphere of *C. arabica* L. was performed as previously stated [12]. The pure isolates were further confirmed by standard microbiological techniques [34]. Pure isolates were stored at -20 °C with 50% glycerol for further study. Primers fD1 (forward, 5′-AGAGTTTGATCCTGGCTCAG-3′) and rP2 (reverse, 5′-ACGGCTACCTTGTTACGACTT-3′), were obtained from Eurofins (India) and used for the amplification of *16S rRNA* gene [35]. A PCR mixture (20 μl) consisting of: 2.5 μl of 10X Mg^2+^ buffer containing 15 mM Mg^2+^, 1 μl of Taq polymerase enzyme (1U/μl), 3 μl of dNTP mixture (3 mM), 1 μl of each primer (10 pM), 2 μl of template DNA (50–100 ng/μl) and 9.5 μl of Milli-Q water was prepared. DNA thermocycler was used for the amplification of the DNA at 94 °C for 3 min, followed by 32 cycles of 30 s at 94 °C, 15 s at 54 °C and 1 min at 72 °C with an extension of 72 °C for 5 min. One μl of PCR product along with standard DNA ladder (1.5 kb) were loaded on a 0.8% agarose gel containing 3 μl of ethidium bromide in 1X TAE buffer and electrophoresed at 100 V for 35 min. The PCR product checked and visualized using a UV transilluminator. The PCR product purified and sequenced at Eurofins Genomics India Pvt Ltd, Bangalore, India.

Using the NCBI website, Basic Local Alignment Search Tool (BLASTn) used for checking the 16S rRNA gene sequences with comparative sequences of reference strains. Mega 6 software used for the construction of phylogenetic tree after the sequences aligned. To carry out phylogenetic analysis, sequences of 16S rRNA of thirteen reference strains that are important for comparison downloaded from NCBI database http://www.ncbi.nlm.nih.gov.

### In vitro antagonistic study of bacteria against pathogens

In vitro antagonistic study of the bacteria was carried out against two fungal pathogens *Colletotrichum gloeosporioids sp.coffee* (ITCC 7131) and *Fusarium oxysporum* (ITCC 3595). Both the cultures were obtained from Indian Type Culture Collection (ITCC), Division of Plant pathology, Indian agricultural research institute, New Delhi 110012 (India).

Potato dextrose agar used for dual culture during the antagonistic studies of bacteria against fungal pathogens*.* Percent fungal radial growth inhibition was calculated as stated below [12].

$$ \mathrm{Fungal}\ \mathrm{mycelial}\ \mathrm{growth}\ \mathrm{inhibition}=\left[\left(\mathrm{C}\hbox{-} \mathrm{T}\right),/,\mathrm{C}\right]\times 100 $$

Where,

$$ \mathrm{T} = \mathrm{fungal}\ \mathrm{radial}\ \mathrm{mycelial}\ \mathrm{growth}\ \mathrm{during}\ \mathrm{dual}\ \mathrm{culture}\ \left(\mathrm{Bacteria} + \mathrm{fungus}\right) $$

$$ \mathrm{C} = \mathrm{fungal}\ \mathrm{radial}\ \mathrm{mycelial}\ \mathrm{growth}\ \left(\mathrm{without}\ \mathrm{antagonistic}\ \mathrm{bacteria}\right). $$

### Experimental design and bioassay

The experiment involved only one factor (the rhizobacteria antagonist). In the experiment *Bacillus sp* BT42, used as it showed the highest reduction of radial mycelial growth of both *C. gloeosporioides* and *F. oxysporum*. Healthy C*offea arabica* L. seeds collected from selected healthy coffee plants. First, the exocarp of the coffee fruits was removed and kept in water at 30 °C for 24 h with the other fruit part (mesocarp and endocarp) then mesocarp was removed by washing them and left on a tray to dry [36]. Surface sterilization of coffee seeds was carried out as reported elsewhere [37]. Two hundred sixteen surface sterilized coffee seeds randomly distributed in four groups: untreated (36 seeds), rhizobacteria treated (36 seeds), rhizobacteria + fungi (72 seeds) and fungi (72 seeds) for the experiment. The bacterium grown in nutrient broth under shaking condition of 150 rpm for 18–20 h (1 X 10^8^ cells/ml) at 30 °C were used for coating seeds. The surface sterilized seeds inoculated in the liquid culture of bacteria and dried in aseptic condition under laminar flow hood for 30 min. In the same manner, spore suspensions of *C. gloeosporioides* (1 X 10^4^ spores/ml) inoculated and dried under laminar flow hood for 30 min. Similarly, bacteria and fungi surface coated on the seed of *C. arabica* L., in the same manner, one after the other. Surface sterilized seeds without any treatment taken as controls.

Seeds in each group (bacterial treated, fungi infected, fungi + bacteria treated and untreated) placed on pre-sterilized Whatman filter paper in separate Petri plate (three replicates). Plates with the coffee seeds incubated at 30 + 1 °C, dampness of the seeds maintained by spraying 1–1.5 ml distilled water on filter paper as necessary and any physiological changes in each group of seeds inspected and recorded daily. The experiment carried out in completely randomized design under controlled conditions. The experiment performed in three replicates.

### Studies of plant growth promoting traits

#### Qualitative and quantitative test for Siderophore production

Rhizobacterium was grown in iron-free medium (K_2_HPO_4_, 6.0 g L^-1^; KH_2_PO_4_, 3.0 g L^-1^; MgSO_4_7H_2_O,0.2 g L^-1^;(NH_4_)_2_SO_4_,1.0 g L^-1^; and Succinic acid 4.0 g L^-1^, pH 7.0) and incubated for 48 h at 30 °C with constant shaking of 150 rpm [38]. After 48 h of incubation, the fermented broth centrifuged at 10,000 rpm for 15 min and supernatant were taken and checked for the presence of siderophore. For estimation of siderophore, 0.5 ml of supernatant was mixed with CAS reagent and absorbance was calculated at 630 nm [38]. Percent siderophore units calculated using the formula:

$$ \%\ \mathrm{s}\mathrm{iderophore}\ \mathrm{units} = \left(\mathrm{A}\mathrm{r}\hbox{-} \mathrm{A}\mathrm{s}\right)/\mathrm{A}\mathrm{r} \times 100 $$

Where, Ar = absorbance of reference at 630 nm (CAS reagent) and As = absorbance of the sample at 630 nm.

#### Test for phytohormones production

For the production of IAA, purified bacterium isolate was grown in Luria-Bertani (LB) broth under shaking condition (150 rpm) around the clock at 30 °C. Overnight grown culture centrifuged at 10,000 rpm for 15 min and the supernatant collected. To the supernatant (app. 2 ml) two drops of O-phosphoric acid added; the manifestation of pink color indicates IAA production by the rhizobacteria isolates. Quantitative estimation of IAA was done colorimetrically [39].

For the production of Gibberellic acid, bacterium isolate was grown in 100 ml nutrient broth at 30 °C for 48 h. The growth of bacterium was monitored by measuring turbidity at 600 nm. For extraction of Gibberellic acid, 50 ml of the culture was taken and centrifuged at 7500 rpm for 10 min. The supernatant was collected and pH was adjusted to 2.5 using 37% HCl. Supernatant extracted using ethyl acetate in 1:1 volume ratio [40]. Gibberellic acid quantitatively estimated by using a UV spectrophotometer at 254 nm [41].

#### Test for ammonia production

Rhizobacterium isolate grown in peptone water for 4 days at 30 °C in 50 ml test tubes. In each test tube containing bacterial isolates, 1 ml of Nessler’s reagent added. The appearance of a faint yellow color is evidence of weak reaction and deep yellow to brownish color was confirmation of strong reaction [42].

#### Test for zinc and phosphate solubilization

Phosphate solubilizing ability of bacterium isolate was determined by the inoculation of overnight grown bacterial isolates on pre-solidified specific medium for phosphate solubilization test [43]. The rhizobacterium incubated for 96 h at 30 °C and any clear zone around the rhizobacterium colonies indicated phosphate solubilization. Quantitative determination of phosphate solubilizing activity was performed calorimetrically [44]. Zinc solubilization was performed by plate assay using modified Pikovskaya agar [45]. The rhizobacteria isolates were inoculated into a medium consisting of: ammonium sulfate (1 g L^-1^), dipotassium hydrogen phosphate (0.2 g L^-1^), glucose (10.0 g L^-1^), magnesium sulfate (0.1 g L^-1^), potassium chloride (0.2 g L^-1^), Yeast (0.2 g L^-1^), distilled water (1000 ml), pH 7.0 and 0.1% insoluble zinc compounds (ZnO, ZnCO_3_ and ZnS). The rhizobacteria isolate grown in this medium for 48 h at 28 °C. The clear zone around the colony indicated solubilization of insoluble zinc compounds.

#### Qualitative test for 1-Aminocyclopropane-1-carboxylate (ACC) deaminase

ACC deaminase production was checked using Dworkin and Foster (DF) minimal salts medium [46]. To the pre-solidified DF minimal salts medium 3 mM ACC solution sprayed and allowed to dry in aseptic condition for 10 min then bacterial isolates inoculated. After 48 h of incubation at 30 °C, any growth of bacteria on the media was considered as ACC deaminase production [47].

### Studies of mechanisms of inhibition of pathogenic fungi

#### Lytic enzymes production

##### Test for Production of β-1, 3 glucanases, and chitinase

Test for the production of β-1, 3 glucanases was performed using laminarin as the only carbon source for growth of bacteria. Accordingly, the isolates were inoculated on media containing Na_2_HPO_4_ (6 g L^-1^), KH_2_PO_4_ (3 g L^-1^), NH_4_Cl (0.5 g L^-1^), yeast extract (0.05 g L^-1^), Agar (15 g L^-1^) and 0.05% laminarin, (Sigma) and incubated at 30 °C for 48 h. After 48 h of incubation, the clear zones obtained. To visualize clearly, plates were flooded with a mixture of 0.666% KI and 0.333% Iodine and isolates showing yellow clear zone around the bacterial colony confirmed as the production of β-1, 3 glucanases.

For the checking of chitinase production, colloidal chitin was prepared from chitin powder (Hi-Media) for the preparation of the solid media. The compositions of solid media were colloidal chitin 1% (w/v), Na_2_HPO_4_ (6 g L^-1^), NaCl (0.5 g L^-1^), KH_2_PO_4_ (3 g L^-1^); NH_4_Cl (1 g L^-1^), yeast extract (0.05 g L^-1^) and agar (15 g L^-1^). The bacteria isolates were checked for their production of chitinase by observation of clear zone around the colonies after five days incubation at 30 ± 1 °C [48].

##### Test for Production of Protease and Lipase

Test for protease production was done using the protease specific medium as earlier described [49]. Similarly, the rhizobacteria isolates were checked for lipase enzyme using lipase media [2]. This media contains calcium chloride 0.1 g, Peptone 10 g, sodium chloride 5 g, Agar 15 g, distilled water 1 Liter, 10 ml sterile Tween 20. The bacterial isolates streaked on this medium and incubated at 27 °C for 48 h, the clear zone around the bacterial colonies show the activity of lipase enzyme.

### Production of antifungal compound from isolate BT42

The test for antifungal activity of the culture filtrate was done as described elsewhere with modifications [50, 51]. Briefly, to check the antifungal activity of culture filtrate from strain BT42, a single colony of the isolate inoculated into Luria-Bertani (LB) broth and incubated for 120 h under shaking condition of 150 rpm at 30 °C. During the incubation period, 5 ml samples taken from flasks at different time intervals and centrifuged at 12,500 rpm for 10 min at 4 °C. The cell pellet removed and supernatant filtered through a membrane filter (25 mm) to remove any suspended cell. The collected sample tested for the antifungal activity by agar well diffusion method using 20 μl of the culture filtrate and sterile broth as a control on Potato dextrose agar spread with a spore suspension of pathogenic fungi (1 X 10^4^ spores/ml). The plates incubated at 28 °C for 3 days.

Extracellular metabolites extracted from the 48 h grown culture filtrate using ethyl acetate. Accordingly, an equal amount of ethyl acetate added to the culture filtrate and both phases collected and concentrated to dryness. Active compounds obtained from both the phases subjected to the antifungal activity bioassay after dissolving them in methanol

### Purification, identification and characterization of antifungal compound

The crude extract dissolved in methanol was separated by TLC on silica gel plates (20 × 20 cm, 0.5 mm thick, G), developed in Isopropanol: ammonia: water (10:1.5:1, v/v) as the mobile phase. The TLC plate was visualized under UV transilluminator. Each specific band corresponding to specific metabolite eluted by carefully scraping from the TLC plate, suspended in methanol and checked for antifungal activity. The antifungal compound was once again subjected to TLC using the same as well as different solvent systems (chloroform: methanol 90:10; benzene: acetic acid 95:05; ammonia: methanol: chloroform 0.5:1:8.5) as stated above to check its purity. The purified compound characterized and identified using FTIR and LC-MS analysis. The alkaloid nature of the purified antifungal compound was also confirmed by a qualitative test using Dragendorff’s reagent and glyoxylic-sulphuric acid test specific for indole derivatives as described elsewhere [52, 53].

### Statistical analysis

IBM SPSS Statistics software version 19 was used to analyze the data related to correlations of fungal radial mycelial growth inhibition and lytic enzyme production. Similarly, one-way ANOVAs was used to compare the mean difference between treatments, and the level of significance was set at *P* < 0.05.

## Abbreviations

ACC: 1-Aminocyclopropane-1-carboxylate
BLAST: Basic Local Alignment Search Tool
*C. arabica*: *Coffea arabica*
*C. gloeosporioides*: *Colletotrichum gloeosporioides*
CAS: Chrome Azul S
DF: Dworkin and Foster
*F. oxysporum*: *Fusarium oxysporum*
FTIR: Fourier transform infrared spectroscopy
IAA: Indole-3-acetic acid
ITCC: Indian Type Culture Collection
LC-MS: Liquid chromatography-mass spectrometry
MS: Mass spectrometry
PDA: Photodiode anode
PGPR: Plant growth promoting rhizobacteria
TLC: Thin layer chromatography
